# Incidence and risk factors of depression after diagnosis of lung cancer: A nationwide population-based study

**DOI:** 10.1097/MD.0000000000006864

**Published:** 2017-05-12

**Authors:** Ming-Szu Hung, I-Chuan Chen, Chuan-Pin Lee, Ru-Jiun Huang, Pau-Chung Chen, Ying-Huang Tsai, Yao-Hsu Yang

**Affiliations:** a Division of Thoracic Oncology, Department of Pulmonary and Critical Care Medicine, Chang Gung Memorial Hospital, Chiayi Branch, Puzi City; b Department of Medicine, College of Medicine, Chang Gung University, Taoyuan; c Department of Respiratory Care, Chang Gung University of Science and Technology, Chiayi Campus; d Department of Emergency Medicine, Chang Gung Memorial Hospital, Chiayi Branch; e Department of Nursing, Chang Gung University of Science and Technology, Chiayi Campus; f Center of Excellence for Chang Gung Research Datalink, Chang Gung Memorial Hospital, Chiayi; g Institute of Occupational Medicine and Industrial Hygiene, National Taiwan University College of Public Health; h Department of Environmental and Occupational Medicine, National Taiwan University Hospital and National Taiwan University, College of Medicine, Taipei; i Department of Respiratory Care, College of Medicine, Chang Gung University, Taoyuan; j Department of Traditional Chinese Medicine, Chang Gung Memorial Hospital, Chiayi Branch, Puzi City; k School of Traditional Chinese Medicine, College of Medicine, Chang Gung University, Taoyuan, Taiwan ROC.

**Keywords:** depression, lung cancer, nationwide

## Abstract

This study aimed to explore the incidence and risk factors of depression after lung cancer diagnosis. Using the Taiwan National Health Insurance Research Database (NHIRD), incidences and risk factors of depression in lung cancer and nonlung cancer cohorts were analyzed.

From 1998 to 2006, a total of 22,125 patients were included in each matched cohort of lung cancer and nonlung cancer patients from NHIRD. The incidence of depression was higher in the lung cancer cohort than in the nonlung cancer cohort (1545.8 vs 1366.6 per 100,000 person-years). An increased risk of depression was observed in the lung cancer cohort [adjusted hazard ratio (aHR): 1.16, 95% confidence interval (95% CI): 1.01–1.34, *P* = .0377]. In lung cancer patients, age ≤50 years (aHR: 2.72, 95% CI: 2.02–3.66, *P* *<* .0001), age 50 to 69 years (aHR: 2.34, 95% CI: 1.87–2.94, *P* *<* .0001), female gender (aHR: 1.50, 95% CI: 1.26–1.80, *P* *<* .0001), coronary artery disease (CAD) (aHR: 1.40, 95% CI: 1.08–1.82, *P* = .0113), and operation (aHR: 1.78, 95% CI: 1.46–2.16, *P* < .0001) were associated with an increased risk of depression. In addition, higher incidences of emergency room (ER) visit (4.76 vs 2.82, per person-year) and admission (5.73 vs 4.33, per person-year) were observed in lung cancer patients with depression than those without depression.

Our results showed that early surveillance and intervention of depression should be advocated after a diagnosis of lung cancer.

## 1 Introduction

Lung cancer is the most common cause of cancer death worldwide.^[1]^ Most patients are diagnosed with advance stages of lung cancer. Despite treatment, the 5-year survival rate for lung cancer remains low.^[2]^ In patients with lung cancer, several physical signs and symptoms, including coughing, wheezing, weight loss, insomnia, fatigue, and chest pain, can disturb the quality of life and cause depressive disorder.^[3]^

The prevalence rate of depression has been reported to be 12.4% in lung cancer patients.^[4]^ The association of depression with a negative impact on patients’ quality of life,^[5]^ decision making on cancer treatment,^[6]^ care giver distress,^[7]^ and increased mortality^[8]^ has been reported among lung cancer patients.

However, previous studies about lung cancer and depression are limited by small sample sizes and nonrandomized cross-sectional designs. In addition, most studies are designed to evaluate health-related quality of life using screening instruments. The risk of depression diagnosed by clinical physicians after diagnosis of lung cancer was rarely explored, as seen in the nationwide database.

In this study, a nationwide population-based retrospective cohort study was designed. The risk of depression diagnosed by clinical physicians after diagnosis of lung cancer, risk factors of depression, and impact of depression on medical attendance in lung cancer patients were evaluated.

## 2 Methods

### 2.1 Data source

Data were sourced from the Taiwan National Health Insurance Research Database (NHIRD). National Health Insurance (NHI) is a compulsory universal program for all residents in Taiwan, and the NHIRD is a comprehensive health care database that covers nearly the entire 23.7 million population of this country. Databases were used for admissions and outpatient visits, including information on patient characteristics such as sex, date of birth, date of admission, date of discharge, dates of visits, and up to 5 discharge diagnoses or 3 outpatient visit diagnoses. Diagnosis was made according to the International Classification of Diseases, Ninth Revision, Clinical Modification (ICD-9-CM) codes. Comprehensive utilization and enrollment information for all patients with “catastrophic illnesses” was also included in this database. This study was approved by the Ethics Review Board of Chang Gung Memorial Hospital, Chiayi Branch, Taiwan (IRB No. 201600067B1). This study adhered to strict confidentially guidelines, in accordance with regulations regarding personal electronic data protection. The data in this study were analyzed anonymously. The requirement for informed consent was waived by the institution review board.

### 2.2 Study cohorts

**Figure 1:**
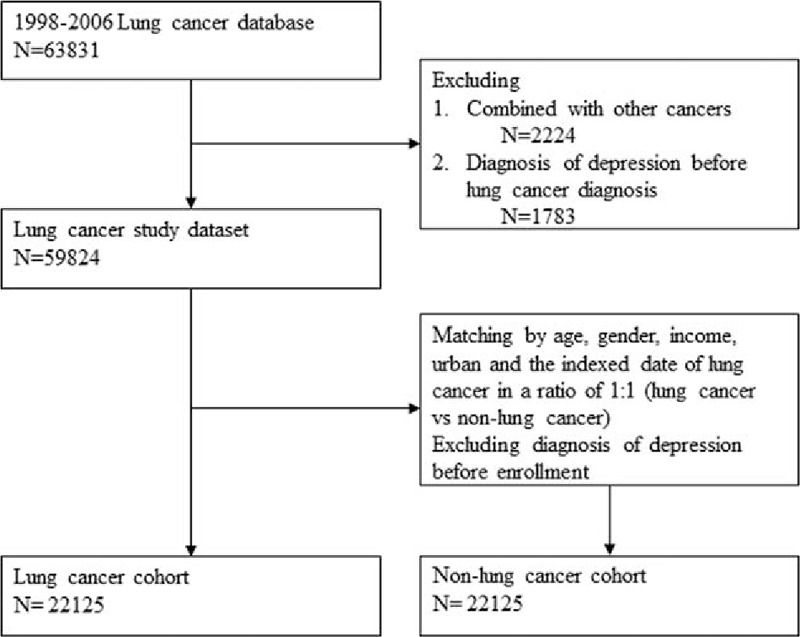
Flowchart of the patient enrollment process of lung cancer cohort and matched nonlung cancer cohort.

We identified all patients with a primary diagnosis of lung cancer (ICD-9-CM 162) for the first time between January 1, 1998, and December 31, 2006, from NHIRD. Those patients with lung cancer diagnosis before January 1, 1998, were excluded to ensure the first diagnosis of lung cancer. Patients diagnosed with other cancers were also excluded. A comparison cohort was randomly selected from the remaining insured population without lung cancer. For each lung cancer patient, 1 person free of lung cancer was selected and matched with age, gender, income, urban level. For each nonlung cancer patient, an index day was given randomly from the lung cancer cohort that was matched. We also excluded subjects diagnosed with depression before enrollment to identify patients with newly onset depression. After excluding subjects with a history of depression at the baseline, we identified 22,125 patients with lung cancer and 22,125 subjects in nonlung cancer cohort for further analysis (Fig. 1). All subjects were followed up to the end of 2008 to measure the incidence of depression. Patients were diagnosed to have depression if they had at least 2 treatment claims for depression in outpatient visits within 1 year or hospitalization with depression (ICD-9-CM 296.2, 296.3, 300.4, or 311) during the follow-up period.

### 2.3 Demographic variables and comorbidities

Age, gender, income for estimating insurance payment, and urbanization of the subject's residential area were included in the demographic variables in this study. Four levels of monthly incomes were determined: NT$0, NT$1 to $15,840, NT$15,841 to $25,000, and ≥NT$25,000. Urbanization levels in Taiwan were divided into 4 levels according to the Taiwan NHRI publications, with level 1 referring to the most-urbanized communities and level 4 to the least urbanized.^[9]^ Hypertension (ICD9-CM 401–405), arthritis (ICD9-CM 715, 716.90), diabetes (ICD9-CM 250), heart disease (ICD9-CM 410–429), chronic kidney disease (ICD9-CM 585), and cancer (ICD9-CM 140–208) were included in the baseline comorbidities for each subject. Data of lung cancer staging are not supplied in the NHIRD. As operation remains the standard treatment for early-stage lung cancer,^[10]^ operation was included as a representative factor of early-stage lung cancer for adjustment of hazard ratio (HR) of depression in lung cancer versus nonlung cancer patients in our study. Brain metastasis and treatments including chemotherapy (CT), radiotherapy (RT), and epidermal growth factor receptor-tyrosine kinase inhibitor (EGFR-TKI) were also included.

### 2.4 Statistical analysis

The differences in demographic characteristics and comorbidities between the lung cancer patients and the comparison cohort were examined by χ^2^ test. As the chance of depression could be confounded by competing risk of mortality, multivariable analyses and subgroup analyses using HRs were calculated with modified Cox proportional hazards models with competing risk event after adjusting for age, gender, income, urban level, diabetes, hypertension, stroke, chronic obstructive pulmonary disease (COPD), arthritis, brain metastasis, operation, CT/RT, and EGFR-TKI for lung cancer. Incidence rates of depression were analyzed as number of cases per 100,000 person-years (PYs) and ER visits, and ward admissions were calculated for each 1 PY. All of these analyses were conducted using SAS statistical software (Version 9.4; SAS Institute, Cary, NC).

## 3 Results

### 3.1 Demographic characteristics and comorbidity between the lung cancer and nonlung cancer cohorts

A total of 59,824 lung cancer patients were included in our study from 1998 to 2006. After being matched with age, gender, income, and levels of urbanization, 22,125 patients were enrolled in both lung cancer and nonlung cancer cohorts. In the lung cancer cohort, significantly higher portions of depression (*P* = .0228), Charlson comorbidity index (CCI) (*P* < .0001), diabetes (*P* < .0001), hypertension (*P* < .0001), stroke (*P* < .0001), CAD (*P* < .0001), COPD (*P* < .0001), and arthritis (*P* < .0001) were observed than in the nonlung cancer cohort (Table 1). The median follow-up period for the nonlung cancer matched cohort was 6.36 [interquartile range (IQR): 4.87] and 0.96 (IQR: 1.81) years for the lung cancer cohort.

**Table 1:**
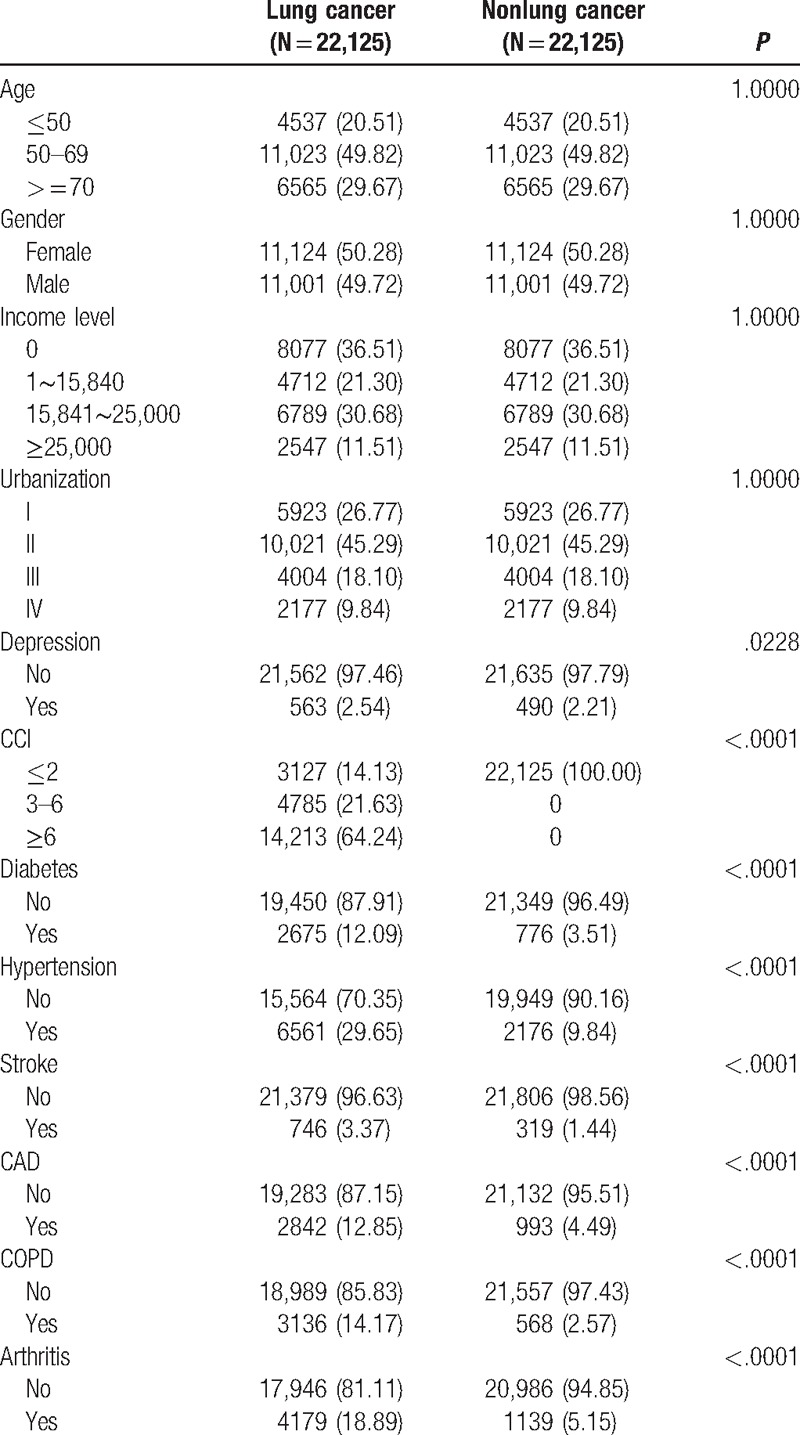
Demographic status and comorbidity compared between cohorts with and without lung cancer.

### 3.2 Incidence and relative risk of depression among lung cancer cohorts

Of the 22,125 patients in lung cancer and nonlung cancer cohorts followed up, the development of depression was observed in 563 patients in the lung cancer and 490 patients in the nonlung cancer cohort. The incidence of depression was higher in the lung cancer cohort than in the nonlung cancer cohort (1545.8 vs 1366.6 per 100,000 PYs). After adjustment of age, sex, income, urbanization, diabetes, hypertension, stroke, CAD, COPD, arthritis, and operation for lung cancer, an increased risk of depression was also observed in the lung cancer cohort with an adjusted hazard ratio (aHR) of 1.16 (95% CI: 1.01–1.34, *P* = .0377) (Table 2).

**Table 2:**
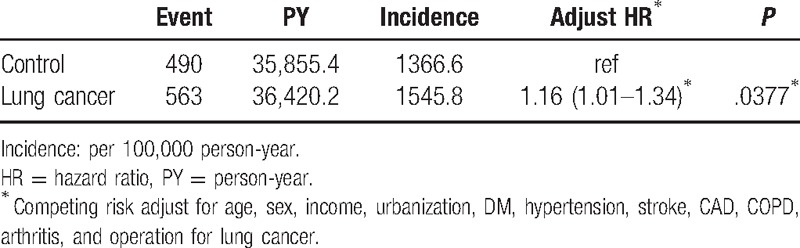
Crude and adjusted hazard ratios of depression for lung cancer patients compared with nonlung cancer control.

### 3.3 Comparison of the HR of depression stratified by age, gender, and comorbidities between lung cancer and nonlung cancer cohorts

The risk of depression in lung cancer and nonlung cancer cohorts was then compared and stratified by age, gender, and comorbidities. In the univariate analysis, an increased risk of depression was observed in lung cancer patients with age ≤50 years, male gender, without diabetes, without hypertension, without stroke, without CAD, with or without COPD, and without or without arthritis. In the multivariate analysis, after adjustment with age, gender, diabetes, hypertension, stroke, CAD, COPD, arthritis, operation, brain metastasis, CT/RT, and EGFR-TKI, an increased risk of depression was observed in lung cancer patients with age ≤50 years (aHR: 4.70, 95% CI: 2.92–7.56, *P* < .0001), age 50 to 69 years (aHR: 1.40, 95% CI: 1.09–1.80, *P* < .0086), male gender (aHR: 1.49, 95% CI: 1.18–1.89, *P* *=* .0008), female gender (aHR: 1.88, 95% CI: 1.40–2.54, *P* < .0001), without diabetes (aHR: 1.63, 95% CI: 1.34–1.98, *P* < .0001), with or without hypertension (aHR: 1.51, 95% CI: 1.02–2.26, *P* = .422; aHR: 1.57, 95% CI: 1.27–1.95, *P* < .0001), without stroke (aHR: 1.59, 95% CI: 1.32–1.92, *P* < .0001), with or without CAD (aHR: 1.92, 95% CI: 1.11–3.32, *P* < .0197; aHR: 1.51, 95% CI: 1.23–1.85, *P* < .0001), with or without COPD (aHR: 2.49, 95% CI: 1.04–5.94, *P* = .0400; aHR: 1.57, 95% CI: 1.29–1.91, *P* < .0001), and with or without arthritis (aHR: 2.05, 95% CI: 1.13–3.72, *P* = .0179; aHR: 1.55, 95% CI: 1.27–1.90, *P* < .0001) (Table 3).

**Table 3:**
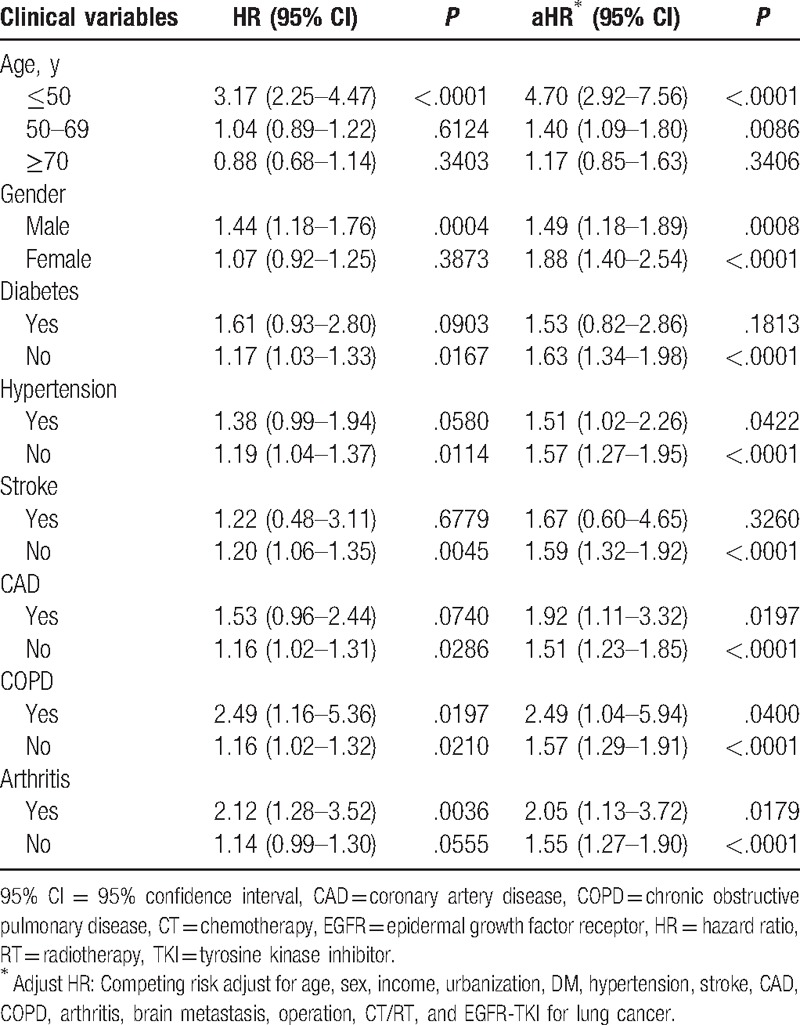
Subgroup analysis based on different age, gender, and comorbidity for the risk of depression in study cohort.

### 3.4 Risk factors for depression in the lung cancer cohort

Risk factors for depression were then analyzed in the lung cancer cohort. In the univariate analysis, an increased risk of depression was observed in patients with age ≤50 years, age 50 to 69 years, female gender, level II urbanization, CAD, and operation. The multivariate analysis further confirmed that age ≤50 years (aHR: 2.72, 95% CI: 2.02–3.66, *P* *<* .0001), age 50 to 69 years (aHR: 2.34, 95% CI: 1.87–2.94, *P* *<* .0001), female gender (aHR: 1.50, 95% CI: 1.26–1.80, *P* *<* .0001), CAD (aHR: 1.40, 95% CI: 1.08–1.82, *P* = .0113), and operation (aHR: 1.78, 95% CI: 1.46–2.16, *P* < .0001) were independent risk factors for depression in lung cancer patients (Table 4). A lower risk of depression was observed in lung cancer patients with monthly income NT$15,841 to $25,000, brain metastasis, CT+RT, RT, and EGFR-TKI (Table 4).

**Table 4:**
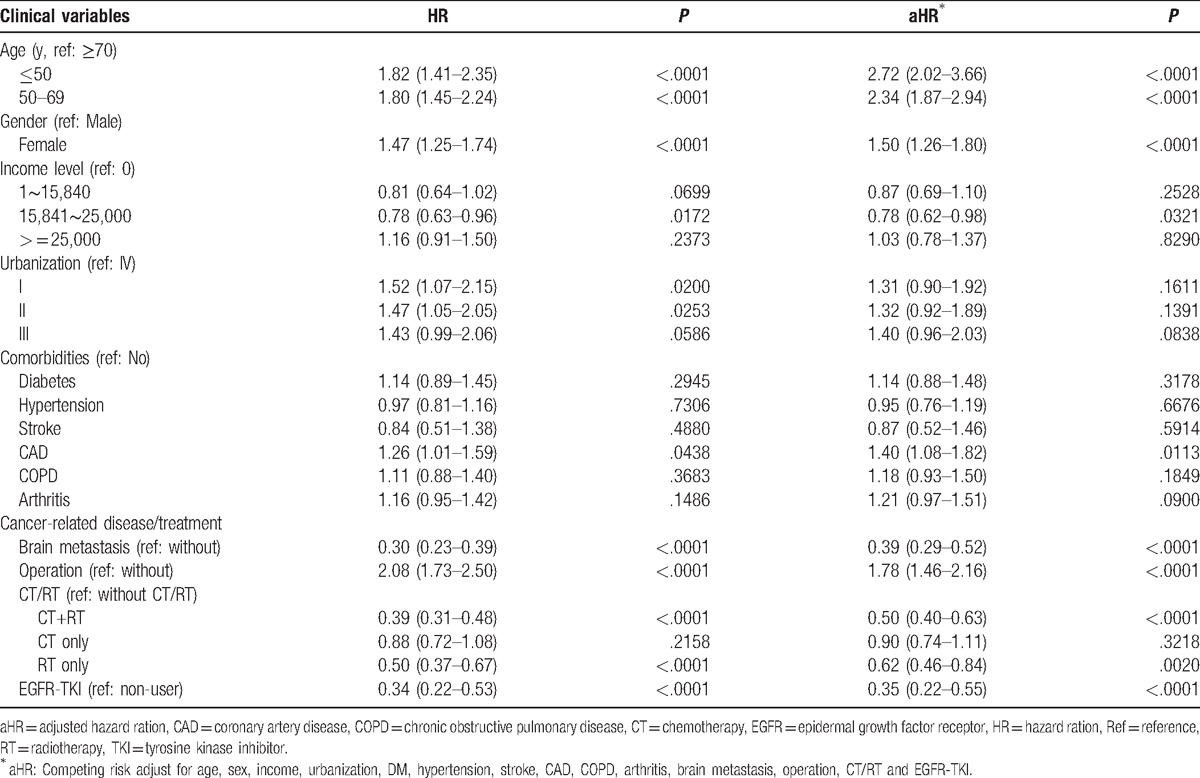
Analysis of risk factors for developing depression among lung cancer patients.

### 3.5 Incidence of ER visit and admission in depression and nondepression lung cancer patients

Of the total of 59,824 lung cancer patients, the incidences of ER visits and admissions to hospital were evaluated. Higher incidences of ER visits (4.76, 95% CI: 4.67–4.86 vs 2.82, 95% CI: 2.81–2.83 per PY) and admissions to hospital (5.73, 95% CI: 5.63–5.83 vs 4.33, 95% CI: 4.32–4.35 per PY) were observed in lung cancer patients with depression than those without depression (Table 5).

**Table 5:**
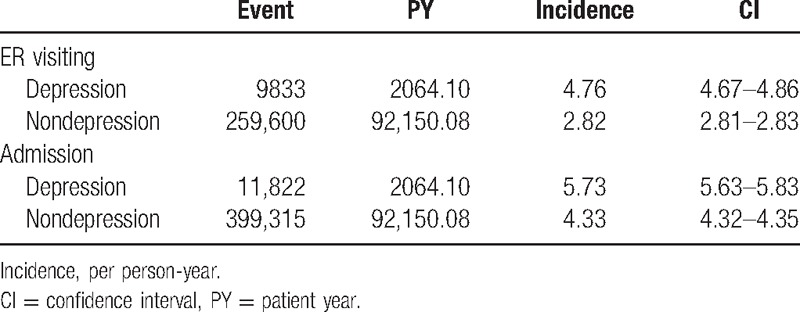
Incidence of ER visiting and admission in depression and nondepression lung cancer patients.

## 4 Discussion

In this retrospective longitudinal cohort study, we observed that higher proportions of comorbidities including diabetes, hypertension, stroke, CAD, COPD, and arthritis were observed in lung cancer patients than the nonlung cancer cohort. The incidence of depression was higher after the diagnosis of the lung cancer than the nonlung cancer cohort. An increased risk of depression was associated with young age, female gender, CAD, and operation in lung cancer patients. Furthermore, higher incidences of ER visits and ward admissions were observed in lung cancer patients with depression.

The development of lung cancer is associated with age and smoking, which are associated with comorbidities.^[11]^ In our study, higher proportions of comorbidities including diabetes, hypertension, stroke, CAD, COPD, and arthritis, which are also associated with age or smoking, were observed in lung cancer patients. Our results are similar to previous studies. Pre-existing diabetes has been reported to increase the risk of lung cancer, especially in female diabetic patients.^[12]^ High blood pressure level is associated with an increased risk of lung cancer in smoking, hypertensive men.^[13]^ Most cancers including lung cancer have been reported to increase the risk of CAD.^[14]^ Stroke has been known as the cerebrovascular complication of lung cancer.^[15]^ Smoking is a common risk factor for COPD and lung cancer and a diagnosis of COPD is strongly associated with a diagnosis of lung cancer.^[16]^ Increased risks of lung cancer as well as other cancers have been reported in patients with rheumatoid arthritis.^[17]^ In our study, the association between the above comorbidities and lung cancer was not further explored. However, our results may still provide additional evidences that in lung cancer patients, further study of the above comorbidities is necessary.

Compared with the nonlung cancer cohort, higher incidence and prevalence rate of depression were observed in the lung cancer cohort in our study, which is similar to previous studies. Higher incidence and prevalence rate of depression in lung cancer patients may be explained by several reasons. Lung cancer is the well-known leading cause of cancer mortality in Taiwan. Because more information has been known about the poor prognosis of lung cancer in the general population, patients may have more fear and frustration after the diagnosis of lung cancer.^[18]^ In addition, patients with lung cancer may have worse quality of life after operation.^[19]^ Side effects of CT or RT for lung cancer patients may cause their psychological distress. Symptoms of lung cancer, such as pain,^[20]^ fatigue, dyspnea, and anorexia,^[21]^ which result in poor physical functioning, psychosocial functioning, and quality of life status, may also predispose to the development of the depressive mood.

In our study, the prevalence rate of depression after a diagnosis of lung cancer is 2.54%. Previous studies reported that the prevalence rate of depression is 9% to 53% in lung cancer patients,^[4,22]^ which is higher than our study. The discrepancy between our study and other studies may be due to the different methods for diagnosis of depression. Unlike previous studies using rating scales or telephone interview,^[23–25]^ clinical diagnosis for depression by attending physicians was used in our study after the diagnosis of lung cancer. Our study may reveal the prevalence rate of depression for patients who need medical intervention by clinical physicians. In addition, patients with previous diagnosis of depression before the initiation of follow-up in both lung cancer and nonlung cancer cohorts were excluded in our study, which may have also resulted in a lower prevalence rate of depression in our study. As lung cancer is a highly malignant disease with high mortality, the median follow-up period in the lung cancer cohort is much less than the nonlung cancer cohort in our study (0.96 vs 6.36 years, respectively). As a result, symptoms of depression may be masked by other symptoms of lung cancer. In addition, most lung cancer patients may not survive long enough to receive clinical evaluation and diagnosis of depression. That is why we used modified Cox proportional hazards models with competing risk event as a statistic model to adjust the influence of mortality.

Young patients and female patients were observed to have higher risks of depression after the diagnosis of lung cancer in our study. Most of the young lung cancer patients were employed. Their workload may contribute to additional emotional distress after diagnosis and during treatment of lung cancer. Female gender has been reported to be associated with higher prevalence of depression,^[26]^ which may be explained by several reasons, including gender role related stressors such as low socioeconomic status, lack of power, role overload, associated psychological attributes such emotion-focused coping styles, interpersonal orientation, and related vulnerability, anxiety, and lowered self-esteem.^[27]^ Moreover, endocrine stress reaction may influence processes leading to depression.^[28]^

Our study showed that operation increased the risk of depression in lung cancer patients. The result is consistent with a recent report and depression after operation may be related to residual symptoms including pain and dyspnea.^[29]^ However, lung cancer patients receiving operation were mostly in earlier stages and were expected live longer to receive psychiatric interventions. On the contrary, patients with brain metastasis, or those receiving CT, RT, and EGFR-TKI treatments were in late stages. Due to short survival, those patients may have less chance to receive psychiatric interventions and a lower risk of depression was observed. Our study showed that more medical attendance to depression should be advocated in this group of lung cancer patients.

Depression has been reported to predict emergency hospital admissions in primary care patients with chronic illness.^[30]^ In our study, higher incidences of ER visits and ward admissions were also observed in lung cancer patients with depression than those without depression. Due to the limitation of the study, the causes of ER visits and ward admissions were not analyzed in our study. However, our study still revealed that more medical attendance was needed in lung cancer patients with depression. As a result, early survey, diagnosis, and intervention for depression are mandatory in lung cancer patients.

There are some limitations in this study. First, data including lung cancer staging, pathology, cancer related symptoms, physical status, smoking status, personal characteristics, and environmental or genetic factors were not supplied in the NHIRD, and all of the potential confounders may be associated with the risk of depressive disorders. Second, the incidence of depression may be underestimated in our study, as only clinically diagnosed depression is enrolled. The severity of depression is not identified in the database.

## 5 Conclusion

Our study shows that higher incidence and prevalence of depression in lung cancer patients, and more medical attendance are required in lung cancer patients with depression. Underdiagnosis of depression is also observed in the clinical practice of lung cancer patients. As a result, early surveillance and intervention of depression should be advocated after the diagnosis of lung cancer, especially in patients with young age, female gender, CAD, and operation.

## Acknowledgment

The authors would like to thank Center of Excellence for Chang Gung Research Datalink for the comments and assistance in data analysis.
